# Markers of Antiviral Response in SLE Patients After Vaccination Against SARS-CoV-2

**DOI:** 10.3390/ijms262010241

**Published:** 2025-10-21

**Authors:** Michał Komorniczak, Katarzyna Aleksandra Lisowska, Barbara Bułło-Piontecka, Alicja Dębska-Ślizień, Anna Wardowska

**Affiliations:** 1 Clinic and Department of Nephrology, Transplantology and Internal Diseases, Faculty of Medicine, Medical University of Gdańsk, 80-214 Gdańsk, Poland; mkomorniczak@gumed.edu.pl (M.K.); bbullo@gumed.edu.pl (B.B.-P.); adeb@gumed.edu.pl (A.D.-Ś.); 2 Department of Rheumatology, Clinical Immunology, Geriatrics and Internal Medicine, Faculty of Medicine, Medical University of Gdańsk, 80-214 Gdańsk, Poland; anna.wardowska@gumed.edu.pl; 3 Department of Pathophysiology, Medical University of Gdańsk, 80-210 Gdańsk, Poland

**Keywords:** systemic lupus erythematosus, lupus nephritis, COVID-19 vaccination, cytokine signatures, type I interferons

## Abstract

Patients with systemic lupus erythematosus (SLE) and lupus nephritis (LN) are at increased risk of severe infections, making effective vaccination strategies essential. While antibody responses to SARS-CoV-2 vaccination have been studied in SLE, less is known about innate immune correlates. Therefore, we evaluated cytokines with a particular emphasis on interferon and chemokine profiles. To fulfill the immunological picture, we also assessed neutralizing antibodies against SARS-CoV-2 variants, lymphocyte subpopulations, and selected gene expression signatures in 33 patients stratified by vaccination status: fully vaccinated (FV, *n* = 23) and partially vaccinated (PV, *n* = 10). Serum analyses showed that FV patients exhibited increased type I (IFN-α2, IFN-β) and type III (IFN-λ1, IFN-λ2/3) interferons, as well as elevated pro-inflammatory cytokines (IL-1β, IL-6, TNF-α, and IL-12p70) and IL-10, whereas neutralizing antibody (Neut. Ab.) titers against wild-type and variant strains, including Omicron, were comparable between groups. Immunophenotyping demonstrated preserved T- and B-cell subset distributions, except for reduced CD8^+^CD197^+^CD45RA^−^ (central memory) T cells in FV patients. ISG15 gene expression was upregulated in the T cells of FV patients. Correlation analyses linked IL-6 with disease activity and IL-8, GM-CSF, IFN-β, IL-10, and Alpha Neut. Ab. with organ damage. Complement C3 correlated inversely with IFN-α2 and IFN-γ, while C4 correlated positively with Alpha and Omicron Neut. Ab. These findings highlight that vaccination in SLE induces distinct interferon and cytokine signatures without consistent enhancement of neutralizing antibodies against SARS-CoV-2, underscoring the importance of integrated immune correlates in assessing vaccine responses in this population.

## 1. Introduction

Vaccination is one of the most transformative achievements in medicine, preventing millions of deaths each year and contributing to the eradication or near-elimination of several pathogens [1,2,3,4]. In immunocompetent hosts, vaccines trigger a coordinated cascade of immune events: innate sensing through pattern-recognition receptors, transient production of type I interferons and pro-inflammatory cytokines, activation of antigen-presenting cells (APCs), and subsequent priming of T and B cells [5,6]. This sequence results in the generation of neutralizing antibodies, long-lived memory B cells, and effector and memory T-cell subsets, which together establish durable protection [7,8].

Patients with systemic lupus erythematosus (SLE), particularly those with lupus nephritis (LN), remain highly vulnerable to severe infections due to intrinsic immune dysregulation and the effects of immunosuppressive therapy. For this reason, international societies such as EULAR (The European Alliance of Associations for Rheumatology) and IDSA (Infectious Diseases Society of America) strongly recommend routine immunization—including influenza, pneumococcal, and human papillomavirus vaccines—as a cornerstone of care, ideally administered during periods of low disease activity and before the initiation of B-cell-depleting therapy [9,10,11]. Consequently, vaccine-induced responses in this population may diverge from the typical pattern observed in healthy individuals, characterized by attenuated neutralizing antibody titers and disproportionate interferon and other cytokine activity. Understanding these differences is crucial for defining reliable immune correlates of protection and for guiding tailored vaccination strategies in SLE [12,13,14].

Evidence accumulated before the coronavirus disease 2019 (COVID-19) pandemic confirmed that inactivated vaccines are safe in SLE, with no meaningful increase in disease activity [12,13,14]. Yet their effectiveness is often attenuated, particularly in patients treated with mycophenolate mofetil, rituximab, or high-dose glucocorticoids [15,16,17,18]. Large COVID-19 vaccine cohorts reinforced these findings: most patients developed protective antibody responses, but titers were consistently lower than in healthy controls, and therapy with B-cell-depleting agents or antimetabolites emerged as the strongest predictors of impaired immunogenicity [16,17,19,20]. Booster doses restored seropositivity in the majority of cases without exacerbating disease activity, confirming that vaccination is both effective and safe [21,22,23].

Most prior studies, however, have focused on humoral outcomes. Far less is known about innate and cytokine-mediated mechanisms that shape early antiviral protection. This gap is critical because dysregulated type I interferon signaling is a defining hallmark of SLE, while pro-inflammatory cytokines such as IL-6, TNF-α, and IL-1β are closely linked to disease activity [24,25,26]. Recent work has suggested that interferons, interferon-stimulated genes, and chemokines such as IP-10 may orchestrate downstream adaptive immunity and influence vaccine effectiveness in SLE patients [19,20,24]. Yet systematic evaluations of these innate immune signatures following vaccination remain scarce.

Against this background, we aimed to characterize antiviral cytokine and chemokine responses, neutralizing antibody titers against SARS-CoV-2 variants, and gene expression signatures in SLE patients with LN according to vaccination status. By integrating serological, cytokine, and transcriptomic profiles, this study aimed to elucidate the immunological correlates of vaccine response in this vulnerable population and to identify markers that may inform tailored vaccination strategies.

## 2. Results

### 2.1. Patient Characteristics

Baseline characteristics were comparable between fully vaccinated (FV, *n* = 23) and partially vaccinated (PV, *n* = 10) patients (Table 1). Median age was 43 years in both groups, with a median disease duration of 19 years in FV and 18 years in PV patients. Disease activity and organ damage, assessed by SLEDAI, SLAM-R, and the SLICC/ACR Damage Index (SDI), did not differ between groups. Laboratory measures, including complement (C3, C4), immunoglobulins (IgG, IgA, and IgM), inflammatory markers (CRP and ESR), and renal function (sCr and eGFR), as well as anthropometric parameters (BMI), were also similar.

### 2.2. Cytokine and Interferon Profiles

**Figure 1:**
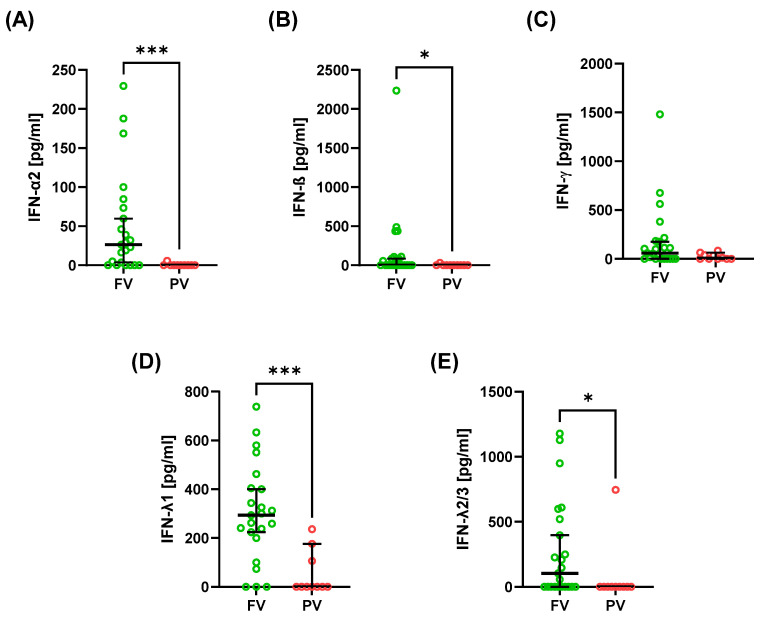
Serum concentration of interferons in SLE patients. Graphs present IFN-α2 (**A**), IFN-β (**B**), IFN-γ (**C**), IFN-λ1 (**D**), and IFN-λ2/3 (**E**). Data are presented as median with 95% confidence interval (CI) according to the U Mann–Whitney test, * *p* < 0.05, and *** *p* < 0.001.

**Figure 2:**
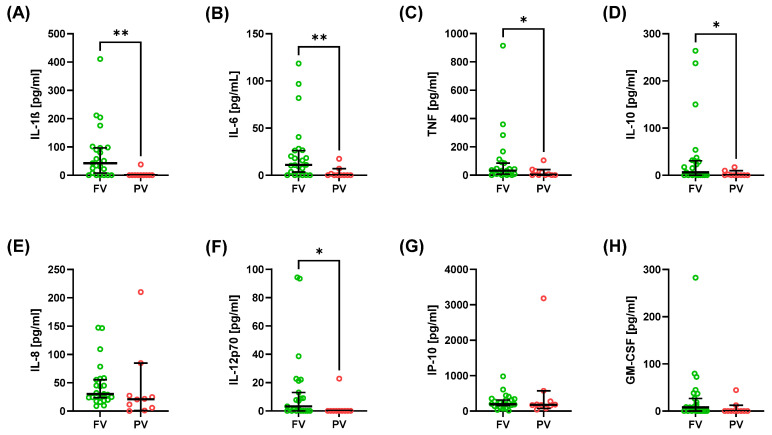
Serum concentration of pro- and anti-inflammatory cytokines and chemokines in SLE patients. Graphs show IL-1β (**A**), IL-6 (**B**), TNF (**C**), IL-10 (**D**), IL-8 (**E**), IL-12p70 (**F**), IP-10 (**G**), and GM-CSF (**H**). Data are presented as median with 95% confidence interval (CI) according to the U Mann–Whitney test, * *p* < 0.05, and ** *p* < 0.01.

Serum cytokine and chemokine profiles differed between FV and PV patients (Table 2; Figure 1 and Figure 2). FV patients exhibited a pronounced interferon signature, with higher type I interferons IFN-α2 (Figure 1A) and IFN-β (Figure 1B) and type III interferons IFN-λ1 (Figure 1D) and IFN-λ2/3 (Figure 1E). Among cytokines, pro-inflammatory mediators were increased in FV–IL-1β (Figure 2A), IL-6 (Figure 2B), TNF-α (Figure 2C), and IL-12p70 (Figure 2F), along with anti-inflammatory cytokine IL-10 (Figure 2D).

In contrast, IFN-γ (Figure 1C), IL-8 (Figure 2E), IP-10 (Figure 2G), and GM-CSF (Figure 2H) showed no significant group differences. Notably, IFN-β was virtually undetectable in PV while present in a subset of FV patients (Figure 1B). Wide interindividual variability was observed for several cytokines (Table 2), particularly TNF-α (Figure 2A) and IL-1β (Figure 2C). This heterogeneity underscores the complexity of immune activation patterns in SLE, despite overall differences between FV and PV groups.

### 2.3. Neutralizing Antibodies Against SARS-CoV-2 Variants

**Figure 3:**
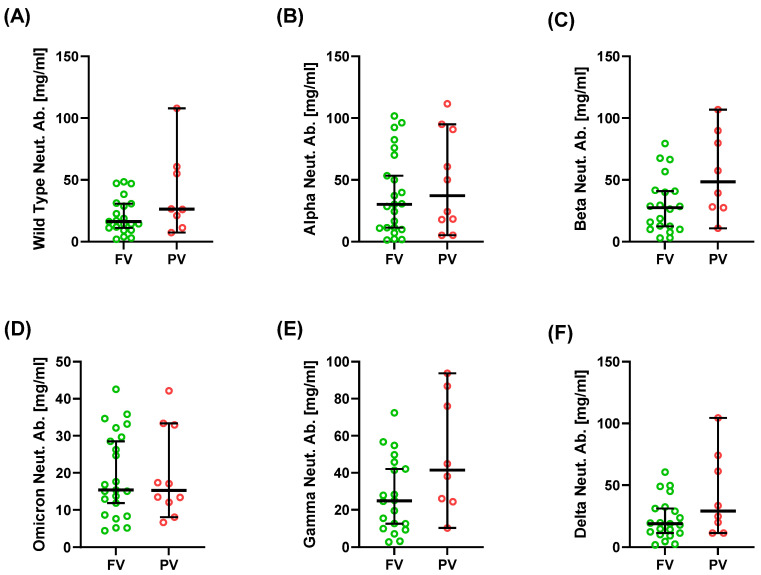
Serum concentration of neutralizing antibodies against SARS-CoV-2 in SLE patients. Graphs show neutralizing antibodies against wild-type S1 (**A**), Alpha S1 (**B**), Beta S1 (**C**), Omicron S1 (**D**), Gamma S1 (**E**), and Delta S1 (**F**). Data are presented as median with 95% confidence interval (CI) according to the U Mann–Whitney test.

Neutralizing antibody (Neut. Ab.) responses did not differ significantly between FV and PV patients (Table 2, Figure 3). Median concentrations of antibodies against wild-type S1 and the Alpha, Beta, Gamma, Delta, and Omicron variants were comparable, with a trend toward higher titers in PV patients for Beta, Gamma, and Delta, although it was not significant (*p* > 0.05). Neutralization of the Omicron variant was low in both groups (median 15.4 vs. 15.3 mg/mL for FV and PV, respectively).

### 2.4. T- and B-Cell Subpopulations

The percentages of T- and B-cell subpopulations are shown in Supplementary Table S2. Overall, the majority of T- and B-cell populations were comparable between FV and PV patients. The only significant difference was observed in the frequency of CD8^+^CD197^+^CD45RA^−^ (central memory) T cells, which was reduced in FV patients. Overall, despite comprehensive immunophenotyping, the cellular composition of adaptive immune subsets appeared largely preserved across vaccination status.

### 2.5. Gene Expression Signatures

T- and B-cell gene expression analyses are shown in Supplementary Table S3. Among T-cell transcripts, only ISG15 expression was significantly higher in FV compared with PV patients (median 0.023 vs. 0.010; *p* = 0.029). No significant differences were observed for other T-cell genes (TBX21, RORC, GATA3, FOXP3, TRIM21, IRF8, IFNG, IL2, and IL10) or B-cell transcripts (APOBEC3G, IRF8, ISG15, BAFF, IL6, and TGFB).

### 2.6. Correlation Analyses

**Figure 4:**
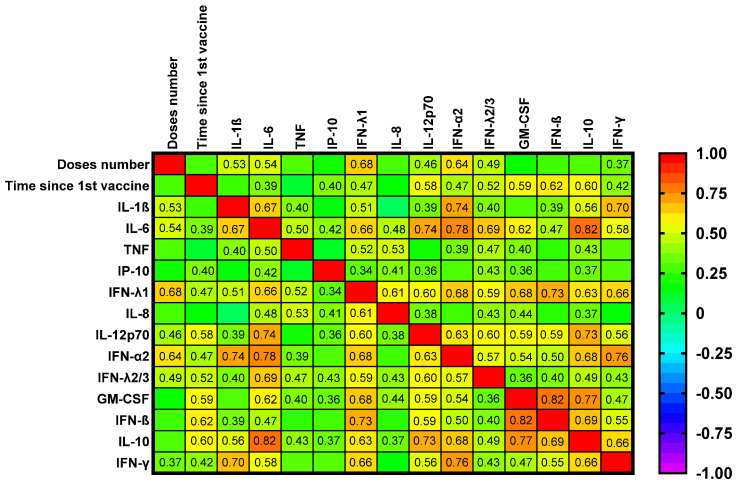
A heatmap of the correlation matrix between serum cytokines/chemokines and the number of vaccination doses and time since the 1st vaccination. The bivariate Spearman Rank Correlation test, the given r values are significant.

**Figure 5:**
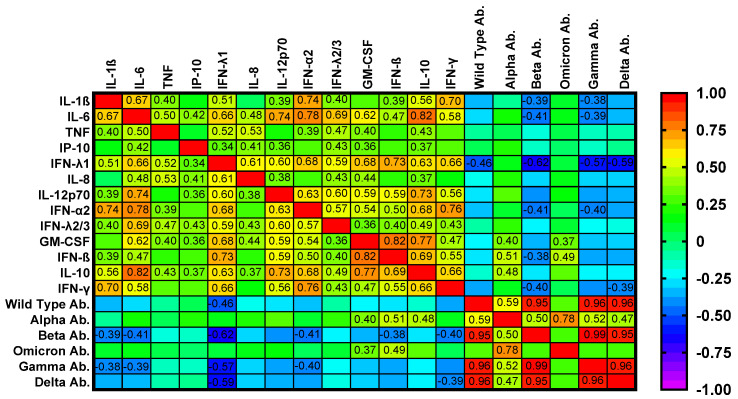
A heatmap of the correlation matrix between serum cytokines/chemokines and neutralizing antibodies against SARS-CoV-2. For the bivariate Spearman Rank Correlation test, the given r values are significant.

Correlation analyses between immunological parameters and clinical or laboratory indices are summarized in Figure 4 and Figure 5 and Supplementary Table S4.

Heatmap analyses (Figure 4 and Figure 5) confirmed strong intercorrelations among cytokines and interferons and showed inverse relationships between selected cytokines and neutralizing responses to Omicron and Delta. Notably, the number of vaccine doses correlated positively with several pro-inflammatory cytokines, including IL-1β, IL-6, IP-10, and IL-12p70, as well as different interferons (Figure 4). The median time from the first vaccine dose was 17.5 months and from the last one 10 months. The time from the first vaccine was positively correlated with serum IL-6, IP-10, IL-12p70, GM-CSF, IL-10, and interferons.

In contrast, neutralizing activity against Omicron and Delta variants correlated negatively with multiple inflammatory mediators (e.g., IL-1β, IL-6, TNF, and IL-12p70), underscoring the interplay between the innate inflammatory tone and the magnitude of humoral protection (Figure 5). These results indicate that cytokine and interferon profiles are linked to clinical indices, complement, and autoantibody levels, and may influence the strength of vaccine-induced neutralizing antibody responses.

Disease activity (SLEDAI) was positively associated with IL-6, while organ damage (SDI) correlated with IL-8, GM-CSF, IFN-β, IL-10, and neutralizing antibodies against the Alpha variant (Supplementary Table S4). Serum complement levels showed distinct associations: C3 was negatively correlated with higher IFN-α2 and IFN-γ, and C4 positively correlated with neutralizing antibodies to Alpha and Omicron. High serum IgG concentrations were associated with high IP-10, and anti-dsDNA titers correlated positively with IP-10 and negatively with Omicron Neut. Ab.

### 2.7. Summary of Results

In this cohort of SLE patients with lupus nephritis, baseline demographic, clinical, and laboratory characteristics were comparable between FV and PV groups. FV patients exhibited consistently higher serum concentrations of type I and type III interferons and selected pro-inflammatory cytokines, whereas neutralizing antibody responses against SARS-CoV-2 variants, including Omicron, were similar across groups. ISG15 expression was elevated in the T cells of FV patients. Several cytokines, interferons, and neutralizing antibodies showed significant associations with disease activity, cumulative organ damage, complement components, and autoantibody titers.

## 3. Discussion

Our study was primarily designed to explore immune profiles within the SLE population, focusing on differences between fully and partially vaccinated patients. This within-cohort comparison was motivated by the ongoing uncertainty regarding the immunogenicity and safety of repeated mRNA vaccination in SLE, particularly in patients receiving immunosuppressive therapy.

Our study shows that in SLE patients with LN, full vaccination against SARS-CoV-2 did not consistently augment neutralizing antibody titers but was accompanied by an increase in type I and III interferon activity and selected pro-inflammatory cytokines, together with upregulation of the ISG15 gene-encoding protein induced by type I IFN in T cells. These data indicate a divergence between humoral and innate correlates of vaccine response in systemic lupus erythematosus, a disorder characterized by interferon-driven pathobiology.

Experience with inactivated vaccines—influenza, pneumococcal, and human papillomavirus—provides context for our findings. Across multiple studies, these vaccines are safe in SLE, with no significant increase in disease activity [14,27]. However, serologic responses are attenuated relative to healthy controls, particularly among patients receiving high-dose glucocorticoids, mycophenolate, or rituximab [14,18]. Higher baseline disease activity and greater immunosuppression further predict reduced immunogenicity [10,26]. Collectively, these observations support the practice of administering vaccines during periods of low disease activity and, when feasible, before the initiation of B-cell-depleting therapy [9,10]. They also suggest that, although vaccines are safe and clinically valuable in SLE, immune responses may not conform to an antibody-centric paradigm.

This divergence can be understood within the interferon-centric SLE biology. Plasmacytoid dendritic cells (pDCs), activated by immune complexes containing nucleic acids via TLR7 and TLR9, drive chronic type I IFN production and a persistent interferon-stimulated gene (ISG) signature [28,29,30,31]. Amplification loops involving neutrophil extracellular traps (NETs), impaired clearance of apoptotic debris, and cGAS–STING (cyclic GMP-AMP synthase–stimulator of interferon genes) signaling further reinforce this state [28]. In such a milieu, vaccination may disproportionately engage interferon and chemokine pathways, which could help explain the dissociation we observed between innate signatures and antibody readouts.

Large prospective cohorts indicate that most SLE patients mount immune responses after mRNA vaccination, although antibody titers are consistently lower than in healthy controls [15,16,17]. Booster doses restore seropositivity in most patients, with up to 93% achieving presumed protective levels after a third dose [22,23] without a clinically meaningful increase in SLE activity. Real-world safety data are likewise reassuring: flares occur in a minority, are usually mild, and are not more frequent than in unvaccinated individuals [32,33,34].

Beyond humoral endpoints, recent studies delineate the cellular compartment. Impaired antibody responses frequently coincide with blunted T-cell function. Izmirly et al. [20] reported that 29% of SLE patients exhibited low humoral responses, and these individuals also demonstrated impaired T-cell function in IFNγ-ELISpot assays. Moyon et al. [19] found that IFN-γ release assays were positive in more than half of responders and correlated with antibody titers. In our cohort, however, circulating IFN-γ levels did not correlate with neutralizing antibodies against any SARS-CoV-2 variant and were not associated with the number of vaccine doses; they were only inversely correlated with complement C3. Collectively, these observations support an assessment of vaccine responses in SLE that integrates humoral, cellular, and innate responses rather than relying solely on serology.

An important finding is the dissociation between neutralizing antibody titers and interferon upregulation. On one hand, type I and III interferons are critical for early antiviral defense and may partially compensate for weak serologic responses, particularly in immunosuppressed patients [31]. On the other hand, IFN signaling is pathogenic in SLE, driving disease activity through the activation of plasmacytoid dendritic cells, TLR (toll-like receptor) engagement, and feed-forward inflammatory loops [28,31]. In our cohort, fully vaccinated patients demonstrated higher concentrations of type I and III interferons, as well as several pro-inflammatory cytokines, compared with partially vaccinated patients; however, clinical activity indices (SLEDAI, SLAM-R) remained comparable between the groups. Moreover, IL-6 correlated positively with SLEDAI, while IFN-β, IL-10, and IL-8 correlated with cumulative organ damage (SDI). In addition, anti-dsDNA titers were uniformly high across the cohort, limiting reliable assessment of their variability; however, the observed association between anti-dsDNA and IP-10 may reflect cytokine-driven bystander activation of autoreactive B cells, as previously proposed in lupus immunopathology [28,31]. The clinical significance of this booster-associated increase in interferon activity is uncertain. While it may contribute to early antiviral protection, it could also potentiate autoimmune pathways in patients with heightened baseline interferon signaling. Our cohort was not powered to detect definitive clinical consequences, and causality cannot be inferred.

Current evidence points to three correlates of vaccine response in SLE: early interferon and cytokine responses, neutralizing antibody titers, and functional T-cell responses. Our results add a transcriptomic dimension; markers such as ISG15 may better reflect interferon activity. However, cellular analyses revealed largely preserved T- and B-cell compartments, except for a reduction in CD8^+^ central memory T cells in fully vaccinated patients. No consistent differences were observed across major B-cell subsets. These findings suggest that, while vaccination amplifies interferon-driven signatures, it does not significantly alter the adaptive cellular composition. A composite panel of these measures is likely to describe vaccine responsiveness more accurately than serology alone.

Our study has limitations. The cohort was modest and single-center. We did not analyze functional T-cell responses, which could correlate with antibody outcomes [19,20]. Follow-up was insufficient to evaluate long-term flare risk. Interferon measurement remains technically challenging; ISG signatures do not distinguish type I from type III pathways [31]. Moreover, the absence of a vaccinated healthy control group limits the ability to attribute interferon and cytokine elevations specifically to SLE rather than to normal post-vaccination responses. However, previous studies in healthy individuals demonstrated that interferon activation after mRNA vaccination is transient and of lower magnitude compared with that observed in our cohort [19,20,24,32,35]. Furthermore, treatment imbalance—particularly, the higher prevalence of mycophenolate mofetil (MMF) in the fully vaccinated group—introduces potential confounding that cannot be resolved in this small cohort. MMF and B-cell-depleting agents are well recognized to attenuate vaccine-induced antibody responses in SLE and related autoimmune diseases [15,17,19,20,22], as reflected in current EULAR recommendations [11] that advocate for vaccination during periods of low disease activity and, where feasible, temporarily adjusting immunosuppressive therapy [11]. These regimens primarily blunt B-cell activation while preserving early interferon signaling, which may account for the stronger cytokine and interferon signatures observed despite comparable neutralizing antibody titers. Finally, the lack of an external replication cohort limits generalizability. These limitations do not alter the principal interpretation but underscore the need for larger, multicenter studies with longer follow-up.

Future studies should validate composite immune panels that combine interferon signatures, cytokine profiles (e.g., IP-10), neutralizing antibody titers, and T-cell assays and link these measures to clinical outcomes, such as breakthrough infections, hospitalizations, and lupus flares. Stratification by interferon tone and disease activity before vaccination may inform individualized booster strategies. Pragmatic, prospective evaluations of immunosuppression adjustments—for example, temporary modification of mycophenolate or timing relative to rituximab—are warranted [17]. Ultimately, composite immune correlates, rather than single biomarkers, will be essential to optimize vaccination in SLE patients.

## 4. Conclusions

In SLE patients with lupus nephritis, vaccination against SARS-CoV-2 was associated with the induction of interferon and pro-inflammatory cytokine signatures rather than the consistent augmentation of neutralizing antibodies. Within the IFN-centric biology of SLE, these findings support personalized vaccination strategies guided by integrated innate, humoral, and cellular immune correlates, as confirmed by prospective clinical studies.

## 5. Materials and Methods

### 5.1. Study Design and Patient Population

The study cohort consisted of 33 patients with systemic lupus erythematosus (SLE) and lupus nephritis, all of whom were under the care of the Department of Nephrology, Transplantology, and Internal Medicine at the Medical University of Gdańsk. All patients fulfilled the Systemic Lupus International Collaborating Clinics (SLICC) classification criteria. Disease activity was assessed using the SLE Disease Activity Index (SLEDAI) and the Systemic Lupus Activity Measure-Revised (SLAM-R), and cumulative organ damage was measured using the SLICC/ACR Damage Index (SDI). The results obtained for all scales are presented in Table 1.

Patients gave written informed consent after the study procedures had been fully explained. The study protocol was approved by the Independent Bioethics Committee for Scientific Research (consent no. NKBBN/506-255/2022, date of approval: 2 June 2021). All experiments were conducted in accordance with the Declaration of Helsinki and relevant institutional guidelines and regulations.

Peripheral fasting venous blood was collected from each patient. A total of 20 mL of blood samples were obtained in EDTA tubes for analysis of T- and B-cell subpopulations and gene expression and in anticoagulant-free tubes for serum preparation. Serum was used to determine concentrations of cytokines, chemokines, and antibodies. All serum samples and isolated cells were stored at −80 °C until analysis.

### 5.2. Vaccination Status

Patients were stratified according to vaccination status. The fully vaccinated (FV) group included 23 patients who had received a complete primary series of two doses followed by a Pfizer-BioNTech booster. The partially vaccinated (PV) group comprised 10 patients who had received fewer than three doses (either a single dose or a two-dose series without a booster). The median times from the first and last vaccine doses were 17.5 months and 10 months, respectively. The primary series consisted of either AstraZeneca or Pfizer-BioNTech, whereas all booster doses were Pfizer-BioNTech.

### 5.3. Clinical and Laboratory Assessment

Baseline clinical and demographic characteristics, including age, disease duration, anthropometric measures (body mass index, BMI), and standard laboratory parameters, were collected for all patients. Laboratory data included complement levels (C3, C4), immunoglobulin concentrations (IgG, IgA, and IgM), inflammatory markers (C-reactive protein, CRP, and erythrocyte sedimentation rate, ESR), and renal function parameters (serum creatinine, sCr, and estimated glomerular filtration rate, eGFR, calculated according to the CKD-EPI equation).

### 5.4. Measurement of Cytokines, Chemokines, and Neutralizing Antibodies

Serum concentrations of cytokines and chemokines were measured using the LEGENDplex™ Human Anti-Virus Response Panel 1 (13-plex; BioLegend, San Diego, CA, USA), following the manufacturer’s protocol. The analytes included IFN-λ1, IL-1β, IL-6, TNF-α, IP-10, IL-8, IL-12p70, IFN-α2, IFN-λ2, GM-CSF, IFN-β, IL-10, and IFN-γ.

Neutralizing antibodies against the SARS-CoV-2 spike S1 protein were determined using the LEGENDplex™ SARS-CoV-2 Variants Neutralizing Antibody Panel (6-plex; BioLegend, San Diego, CA, USA). Variants analyzed included Alpha (B.1.1.7), Beta (B.1.351), Gamma (P.1), Delta (B.1.617.2), Omicron (B.1.1.529), and the wild-type strain.

Quantitative fluorescence analysis was performed with a FACSAria III cytometer (Becton Dickinson, Franklin Lakes, NJ, USA). Data were analyzed with the LEGENDplex™ Data Analysis Software (Version 8.0, https://www.biolegend.com/en-us/legendplex#analysis-software, accessed on 12 December 2024).

### 5.5. Phenotypic Analysis of T- and B-Cell Subpopulations Ex Vivo

Samples of 100 μL per tube of blood were transferred for red blood cell (RBC) lysis. Then, cells were washed with PBS (phosphate-buffered saline) buffer and stained with monoclonal antibodies with fluorescent dyes: CD3 FITC, CD24 FITC, CD69 PE, CCR7 (CD197) PE, CD25 PE, IgD PE, CD28 APC, CD38 APC, CD8 APC-Cy7, CD4 V450, CD19 V450, HLA-DR V450, CD27 V500, HLA-DR V500 (BD Pharmingen™, San Diego, CA, USA), CD45RA APC, and CD127 APC (BioLegend, San Diego, CA, USA) (staining panel for T and B cells), for 30 min at room temperature in the dark. Cells were then washed and suspended in 200 μL of PBS for flow cytometric analysis using the FACSVerse instrument (Becton Dickinson, Franklin Lakes, NJ, USA).

### 5.6. Molecular Analysis of T- and B-Cell Gene Expression

Peripheral blood mononuclear cells (PBMCs) were isolated from whole blood by centrifugation using Histopaque^®^-1077 (Sigma Aldrich Inc., Saint Louis, MO, USA), according to the manufacturer’s protocol. PBMCs were further processed for magnetic cell separation of CD19-positive cells (B cells) using Dynabeads™ CD19 Pan B (Thermo Fisher Scientific Inc., Waltham, MA, USA). CD19-negative cells were used to isolate CD3-positive cells (T cells) with Dynabeads™ CD3 (Thermo Fisher Scientific Inc., Waltham, MA, USA). Both separations were performed according to the manufacturer’s protocol. Isolated T and B cells were kept at −80 °C for molecular analysis.

RNA was isolated using the GeneMATRIX Universal DNA/RNA/Purification Kit (EurX, Gdańsk, Poland). The quality of the samples was evaluated using an Epoch Spectrophotometer (Agilent BioTek, Santa Clara, CA, USA). Total RNA was reverse transcribed into cDNA with the NG dART RT Kit (EurX, Gdańsk, Poland). A total of 20 T-cell transcripts (TBX21, RORC, GATA3, FOXP3, TRIM21, ACE2, APOBEC3G, IRF8, ISG15, IFNG, IL4, IL6, TGFB, IL12, IL2, IL1A, IL17A, IL10, IL8, and TNF) and 9 B-cell transcripts (BAFF, APOBEC3G, IRF8, ISG15, IL6, TGFB, IL12, IL10, and TNF) were analyzed with the TaqMan™ Gene Expression Assay (Thermo Fisher Scientific Inc., Waltham, MA, USA). A list of genes is provided in Supplementary Table S1. Real-time PCR was performed using the Maxima Probe qPCR Master Mix (Thermo Fisher Scientific Inc., Waltham, MA, USA) and PikoReal Real-Time PCR System (Thermo Fisher Scientific Inc., Waltham, MA, USA). Reactions were prepared in a total volume of 10 μL. The cycling conditions were as follows: one cycle of 50 °C for 2 min (UDG pre-treatment) and 95 °C for 10 min (initial denaturation), followed by 40 cycles of 95 °C for 10 s (denaturation) and 60 °C for 60 s (annealing). Relative quantities of target genes were determined for unknown samples using the comparative threshold cycle (ΔCT) method and normalized to GAPDH expression.

### 5.7. Phenotypic Analysis of T- and B-Cell Subpopulations by Flow Cytometry

The cytometric data were analyzed using the FlowJo 10 software (Beckton Dickinson, Franklin Lakes, NJ, USA), as previously described [35]. Lymphocytes were selected based on their characteristics. T cells were identified based on their positivity for CD3 antigen and B cells based on CD19. In T cells, helper T cells were identified by CD4 expression and cytotoxic T cells by CD8 expression. Cells expressing different antigens (CD28, CD69, and HLA-DR) were identified within CD4- and CD8-positive subpopulations.

The analysis of surface expression of CD197 (CCR7) and CD45RA in T cells allowed us to analyze T-cell memory compartments: naive T cells (CD197+CD45RA+ cells), central memory T cells (Tcm) (CD197+CD45RA− cells), effector memory T cells (Tem) (CD197−CD45RA− cells), and effector memory re-expressing T cells (Temra) (CD197−CD45RA+ cells). Regulatory CD4+ T cells (Tregs) were identified based on the surface expression of CD25 and CD127 (CD4+CD127−CD25+ cells).

In the B-cells, transitional B cells (TB) were identified as CD24++CD38++ and plasmablasts (PB) as CD24-CD38++CD27+IgD−. In CD38− B cells, double-negative (CD27−IgD−) memory (DNM) B cells, switched memory (SM) B cells (CD27+IgD− cells), non-switched memory (NSM) B cells (CD27+IgD+ cells), and naive B cells (CD27−IgD+ cells) were identified.

### 5.8. Statistical Analysis

Statistical analyses were performed using GraphPad Prism version 9 (GraphPad Software, Boston, MA, USA). Normality of data distribution was assessed using both the Shapiro–Wilk and Kolmogorov–Smirnov tests. For comparisons between two groups, either Student’s *t*-test (for normally distributed variables) or Mann–Whitney U test (for non-normally distributed variables) was applied. Categorical variables were compared using Fisher’s exact test because of the limited sample size and small expected counts in some categories. For multiple-group comparisons, ANOVA with Tukey’s post hoc test or Kruskal–Wallis with Dunn’s correction was used, as appropriate. Correlations were evaluated using Spearman’s rank correlation coefficient.

Data are presented as median with range (minimum–maximum) or *n* (%) in tables and as median with 95% confidence interval (CI) in figures, unless otherwise specified. All tests were two-tailed, and a *p*-value < 0.05 was considered statistically significant.

Heatmap visualizations were generated to depict correlation matrices, with significance determined according to the Spearman Rank Correlation test. Correction for multiple comparisons was applied where indicated.

## Figures and Tables

**Table 1:** Characteristics of SLE patients.

Variable	Fully Vaccinated (*n* = 23)	Partially Vaccinated (*n* = 10)	*p* Value
Age [years]	43.0 (33.0; 71.0)	43.0 (39.0; 71.0)	0.923
Male-to-female ratio	3:20	0:10	0.536
SLE duration [years]	19.0 (2.0; 42.0)	17.5 (6.0; 37.0)	0.985
LN duration [years]	12.0 (1.0; 25.0)	16.5 (1.0; 32.0)	0.451
Body mass index (BMI) [kg/m^2^]	24.6 (16.7; 34.9)	25.3 (16.4; 30.5)	0.862
SLEDAI [points]	6.0 (0.0; 24.0)	8.0 (2.0; 26.0)	0.603
SLAM-R [points]	4.0 (1.0; 9.0)	6.5 (2.0; 14.0)	0.123
SDI [points]	1.0 (0.0; 5.0)	0.5 (0.0; 6.0)	0.603
Complement C3 [g/L]	1.02 (0.59; 1.89)	1.07 (0.76; 1.65)	0.237
Complement C4 [g/L]	0.16 (0.10; 0.29)	0.20 (0.07; 0.29)	0.324
IgG [g/L]	11.5 (7.7; 16.8)	11.8 (6.2; 23.0)	0.819
IgA [g/L]	2.52 (1.56; 4.83)	2.62 (0.00; 3.70)	0.466
IgM [g/L]	0.88 (0.18; 1.87)	0.86 (0.00; 1.86)	0.819
Anti-dsDNA antibodies [IU/mL]	304.8 (0.0; 800.0)	176.0 (0.0; 800.0)	0.564
ESR [mm/h]	10.0 (2.0; 36.0)	18.0 (2.0; 42.0)	0.393
CRP [mg/L]	1.34 (0.00; 6.09)	2.06 (0.40; 13.35)	0.434
sCr [mg/dL]	0.83 (0.53; 3.80)	0.87 (0.66; 3.12)	0.507
eGFR [mL/min/1.73 m^2^]	85.2 (13.0; 90.0)	81.0 (39.0; 90.0)	0.915
Therapy			
Glucocorticoid use, *n* (%)	20 (87%)	8 (80%)	0.627
Prednisone equivalent dose [mg/day]	5 (0; 20)	5 (0; 10)	0.499
MMF use, *n* (%)	17 (74%)	1 (10%)	**0.001**
CNI use (TAC or CsA), *n* (%)	2 (8.7%)	3 (30%)	0.149
Antimalarial therapy, *n* (%)	15 (65.2%)	4 (40%)	0.257

Data are presented as median (minimum; maximum) or *n* (%). **Abbreviations**: BMI, body mass index; CNI, calcineurin inhibitor; CRP, C-reactive protein; CsA, cyclosporin A; ESR, erythrocyte sedimentation rate; eGFR, estimated glomerular filtration rate; LN, lupus nephritis; MMF, mycophenolate mofetil; sCr, serum creatinine; SDI, SLICC/ACR Damage Index; SLEDAI, SLE Disease Activity Index; SLAM-R, Systemic Lupus Activity Measure-Revised; and TAC, tacrolimus.

**Table 2:** Serum cytokines, chemokines, and SARS-CoV-2 neutralizing antibodies in SLE patients.

Variable	Fully Vaccinated (*n* = 23)	Partially Vaccinated (*n* = 10)	*p* Value
IL-1β [pg/mL]	42.83 (0.00; 410.67)	0.00 (0.00; 38.30)	**0.002**
IL-6 [pg/mL]	11.03 (0.00; 118.47)	0.00 (0.00; 17.43)	**0.003**
TNF-α [pg/mL]	31.87 (0.00; 914.89)	3.44 (0.00; 104.41)	**0.046**
IP-10 [pg/mL]	190.20 (11.89; 979.97)	176.72 (37.09; 3180.58)	0.681
IFN-λ1 [pg/mL]	293.78 (0.00; 738.11)	0.00 (0.00; 236.41)	**0.001**
IL-8 [pg/mL]	30.15 (8.77; 147.14)	21.03 (0.00; 210.19)	0.132
IL-12p70 [pg/mL]	3.19 (0.00; 94.33)	0.00 (0.00; 22.77)	**0.014**
IFN-α2 [pg/mL]	26.45 (0.00; 229.38)	0.00 (0.00; 5.77)	**0.001**
IFN-λ2/3 [pg/mL]	104.21 (0.00; 1177.92)	0.00 (0.00; 745.10)	**0.031**
GM-CSF [pg/mL]	7.90 (0.00; 282.67)	0.00 (0.00; 44.38)	0.076
IFN-β [pg/mL]	0.00 (0.00; 2237.08)	0.00 (0.00; 29.89)	**0.045**
IL-10 [pg/mL]	6.36 (0.00; 263.83)	0.00 (0.00; 17.12)	**0.046**
IFN-γ [pg/mL]	59.70 (0.00; 1480.54)	6.15 (0.00; 83.45)	0.086
Alpha Neut. Ab. [mg/mL]	30.25 (1.30; 101.74)	37.20 (5.10; 111.51)	0.570
Beta Neut. Ab. [mg/mL]	27.45 (2.92; 79.38)	48.39 (10.85; 106.78)	0.079
Omicron Neut. Ab. [mg/mL]	15.38 (4.41; 42.56)	15.28 (6.65; 42.15)	0.953
Gamma Neut. Ab. [mg/mL]	24.91 (2.73; 72.34)	41.49 (10.17; 93.70)	0.098
Wild-type Neut. Ab. [mg/mL]	16.14 (1.91; 48.43)	26.30 (7.30; 107.88)	0.164
Delta Neut. Ab. [mg/mL]	18.75 (1.70; 60.62)	29.09 (11.15; 104.45)	0.102

Data presented as median (minimum; maximum). Statistically significant results (*p* < 0.05) are shown in bold according to the U Mann–Whitney test. **Abbreviations**: GM-CSF, granulocyte–macrophage colony-stimulating factor; IFN, interferon; IL, interleukin; IP-10, interferon-γ-induced protein 10; Neut. Ab., neutralizing antibodies; and TNF, tumor necrosis factor.

## Data Availability

The data presented in this study are available on request from the corresponding author.

## Supplementary Materials

The following supporting information can be downloaded at: https://www.mdpi.com/article/10.3390/ijms262010241/s1.

## Abbreviations

The following abbreviations are used in this manuscript:

ACR	American College of Rheumatology
ACE2	angiotensin-converting enzyme 2
APC	allophycocyanin (fluorochrome)
APOBEC3G	apolipoprotein B mRNA editing enzyme catalytic subunit 3G
BAFF	B-cell activating factor (TNFSF13B)
BMI	body mass index
cDNA	complementary DNA
cGAS	cyclic GMP–AMP synthase
CCR7	C–C chemokine receptor type 7 (CD197)
CD	cluster of differentiation
CI	confidence interval
CKD-EPI	Chronic Kidney Disease Epidemiology Collaboration (equation)
COVID-19	coronavirus disease 2019
CRP	C-reactive protein
CXCL10	C-X-C motif chemokine ligand 10 (see IP-10)
ΔCt	delta threshold cycle (comparative Ct)
DNM	double-negative memory (B cells)
eGFR	estimated glomerular filtration rate
EDTA	ethylenediaminetetraacetic acid
ELISpot	enzyme-linked immunospot
ESR	erythrocyte sedimentation rate
EULAR	European Alliance of Associations for Rheumatology
FACS	fluorescence-activated cell sorting
FITC	fluorescein isothiocyanate
FOXP3	forkhead box P3
FV	fully vaccinated
GAPDH	glyceraldehyde-3-phosphate dehydrogenase
GATA3	GATA-binding protein 3
GM-CSF	granulocyte–macrophage colony-stimulating factor
HLA-DR	human leukocyte antigen–DR isotype
IDSA	Infectious Diseases Society of America
IFN	interferon
IgA	immunoglobulin A
IgG	immunoglobulin G
IgM	immunoglobulin M
IGRA	interferon-γ release assay
IL	interleukin
IP-10	interferon-γ–induced protein 10 (CXCL10)
IRF8	interferon regulatory factor 8
ISG	interferon-stimulated gene
LN	lupus nephritis
mRNA	messenger RNA
MWU	Mann–Whitney U (test)
NETs	neutrophil extracellular traps
Neut. Ab.	neutralizing antibodies
NSM	non-switched memory (B cells)
PB	plasmablasts
PBMCs	peripheral blood mononuclear cells
PBS	phosphate-buffered saline
PE	phycoerythrin
PV	partially vaccinated
qPCR	quantitative polymerase chain reaction
RBC	red blood cell
RORC	RAR-related orphan receptor C (RORγt)
RT	reverse transcription
RT-qPCR	reverse-transcription quantitative PCR
S1	SARS-CoV-2 spike protein subunit 1
SARS-CoV-2	severe acute respiratory syndrome coronavirus 2
SDI	SLICC/ACR Damage Index
SLE	systemic lupus erythematosus
SLEDAI	Systemic Lupus Erythematosus Disease Activity Index
SLAM-R	Systemic Lupus Activity Measure–Revised
sCr	serum creatinine
SLICC	Systemic Lupus International Collaborating Clinics
SM	switched memory (B cells)
STING	stimulator of interferon genes
TB	transitional B cells
TBX21	T-box transcription factor TBX21 (T-bet)
Tcm	central memory T cells
Tem	effector memory T cells
Temra	effector memory T cells re-expressing CD45RA
TGFB	transforming growth factor beta (gene)
TLR	toll-like receptor
TNF	tumor necrosis factor (gene)
Tregs	regulatory T cells
TRIM21	tripartite motif-containing 21 (Ro52)
UDG	uracil-DNA glycosylase
WT	wild type
pDCs	plasmacytoid dendritic cells

## References

[1] Shattock A.J., Johnson H.C., Sim S.Y., Carter A., Lambach P., Hutubessy R.C.W., Thompson K.M., Badizadegan K., Lambert B., Ferrari M.J. (2024). Contribution of vaccination to improved survival and health: Modelling 50 years of the Expanded Programme on Immunization. Lancet.

[2] Greenwood B. (2014). The contribution of vaccination to global health: Past, present and future. Philos. Trans. R. Soc. B Biol. Sci..

[3] Riedel S. (2005). Edward Jenner and the History of Smallpox and Vaccination. Baylor University Medical Center Proceedings.

[4] Smith K.A. (2012). Louis Pasteur, the Father of Immunology?. Front. Immunol..

[5] Pollard A.J., Bijker E.M. (2020). A guide to vaccinology: From basic principles to new developments. Nat. Rev. Immunol..

[6] Pardi N., Hogan M.J., Porter F.W., Weissman D. (2018). mRNA vaccines—A new era in vaccinology. Nat. Rev. Drug Discov..

[7] Zimmermann P., Curtis N. (2019). Factors That Influence the Immune Response to Vaccination. Clin. Microbiol. Rev..

[8] Plotkin S.A. (2010). Correlates of Protection Induced by Vaccination. Clin. Vaccine Immunol..

[9] van Assen S., Agmon-Levin N., Elkayam O., Cervera R., Doran M.F., Dougados M., Emery P., Geborek P., Ioannidis J.P.A., Jayne D.R.W. (2011). EULAR recommendations for vaccination in adult patients with autoimmune inflammatory rheumatic diseases. Ann. Rheum. Dis..

[10] Rubin L.G., Levin M.J., Ljungman P., Davies E.G., Avery R., Tomblyn M., Bousvaros A., Dhanireddy S., Sung L., Keyserling H. (2014). Executive Summary: 2013 IDSA Clinical Practice Guideline for Vaccination of the Immunocompromised Host. Clin. Infect. Dis..

[11] Landewé R.B.M., Kroon F.P.B., Alunno A., Najm A., Bijlsma J.W., Burmester G.-R.R., Caporali R., Combe B., Conway R., Curtis J.R. (2022). EULAR recommendations for the management and vaccination of people with rheumatic and musculoskeletal diseases in the context of SARS-CoV-2: The November 2021 update. Ann. Rheum. Dis..

[12] Murdaca G., Orsi A., Spanò F., Puppo F., Durando P., Icardi G., Ansaldi F. (2014). Influenza and pneumococcal vaccinations of patients with systemic lupus erythematosus: Current views upon safety and immunogenicity. Autoimmun. Rev..

[13] Garg M., Mufti N., Palmore T.N., Hasni S.A. (2018). Recommendations and barriers to vaccination in systemic lupus erythematosus. Autoimmun. Rev..

[14] Pugès M., Biscay P., Barnetche T., Truchetet M., Richez C., Seneschal J., Gensous N., Lazaro E., Duffau P. (2016). Immunogenicity and impact on disease activity of influenza and pneumococcal vaccines in systemic lupus erythematosus: A systematic literature review and meta-analysis. Rheumatology.

[15] Yuki E.F.N., Borba E.F., Pasoto S.G., Seguro L.P., Lopes M., Saad C.G.S., Medeiros-Ribeiro A.C., Silva C.A., de Andrade D.C.O., Kupa L.d.V.K. (2022). Impact of Distinct Therapies on Antibody Response to SARS-CoV-2 Vaccine in Systemic Lupus Erythematosus. Arthritis Care Res..

[16] Furer V., Eviatar T., Zisman D., Peleg H., Paran D., Levartovsky D., Zisapel M., Elalouf O., Kaufman I., Meidan R. (2021). Immunogenicity and safety of the BNT162b2 mRNA COVID-19 vaccine in adult patients with autoimmune inflammatory rheumatic diseases and in the general population: A multicentre study. Ann. Rheum. Dis..

[17] Petri M., Joyce D., Haag K., Fava A., Goldman D.W., Zhong D., Xiao S., Milstone A., Magder L.S. (2023). Effect of Systemic Lupus Erythematosus and Immunosuppressive Agents on COVID-19 Vaccination Antibody Response. Arthritis Care Res..

[18] Adawi M., Bragazzi N.L., McGonagle D., Watad S., Mahroum N., Damiani G., Conic R., Bridgewood C., Mahagna H., Giacomelli L. (2019). Immunogenicity, safety and tolerability of anti-pneumococcal vaccination in systemic lupus erythematosus patients: An evidence-informed and PRISMA compliant systematic review and meta-analysis. Autoimmun. Rev..

[19] Moyon Q., Sterlin D., Miyara M., Anna F., Mathian A., Lhote R., Ghillani-Dalbin P., Breillat P., Mudumba S., de Alba S. (2021). BNT162b2 vaccine-induced humoral and cellular responses against SARS-CoV-2 variants in systemic lupus erythematosus. Ann. Rheum. Dis..

[20] Izmirly P.M., Kim M.Y., Samanovic M., Fernandez-Ruiz R., Ohana S., Deonaraine K.K., Engel A.J., Masson M., Xie X., Cornelius A.R. (2021). Evaluation of Immune Response and Disease Status in Systemic Lupus Erythematosus Patients Following SARS–CoV-2 Vaccination. Arthritis Rheumatol..

[21] Tunitsky-Lifshitz Y., Maoz-Segal R., Niznik S., Shavit R., Yahia S.H., Langevitz P., Agmon-Levin N. (2023). The third dose of BNT162b2 COVID-19 vaccine is efficacious and safe for systemic lupus erythematosus patients receiving belimumab. Lupus.

[22] Larsen E.S., Nilsson A.C., Möller S., Voss A.B., Johansen I.S. (2022). Immunogenicity and risk of disease flare after a three-dose regimen with SARS-CoV-2 vaccination in patients with systemic lupus erythematosus: Results from the prospective cohort study COVAC-SLE. Clin. Exp. Rheumatol..

[23] Tan S.Y.S., Yee A.M., Sim J.J.L., Lim C.C. (2022). COVID-19 vaccination in systemic lupus erythematosus: A systematic review of its effectiveness, immunogenicity, flares and acceptance. Rheumatology.

[24] Tang W., Askanase A.D., Khalili L., Merrill J.T. (2021). SARS-CoV-2 vaccines in patients with SLE. Lupus Sci. Med..

[25] Khatri G., Shaikh S., Rai A., Cheema H.A., Essar M.Y. (2022). Systematic lupus erythematous patients following COVID-19 vaccination: Its flares up and precautions. Ann. Med. Surg..

[26] Murdaca G., Orsi A., Spanò F., Faccio V., Puppo F., Durando P., Icardi G., Ansaldi F. (2015). Vaccine-preventable infections in Systemic Lupus Erythematosus. Hum. Vaccines Immunother..

[27] Ioannou Y., Isenberg D.A. (1999). Immunisation of patients with systemic lupus erythematosus: The current state of play. Lupus.

[28] Choi J., Kim S.T., Craft J. (2012). The pathogenesis of systemic lupus erythematosus—An update. Curr. Opin. Immunol..

[29] Postal M., Vivaldo J.F., Fernandez-Ruiz R., Paredes J.L., Appenzeller S., Niewold T.B. (2020). Type I interferon in the pathogenesis of systemic lupus erythematosus. Curr. Opin. Immunol..

[30] Kim J.-M., Park S.-H., Kim H.-Y., Kwok S.-K. (2015). A Plasmacytoid Dendritic Cells-Type I Interferon Axis Is Critically Implicated in the Pathogenesis of Systemic Lupus Erythematosus. Int. J. Mol. Sci..

[31] Caielli S., Wan Z., Pascual V. (2023). Systemic Lupus Erythematosus Pathogenesis: Interferon and Beyond. Annu. Rev. Immunol..

[32] Mok C.C., Chan K.L., Tse S.M. (2022). Hesitancy for SARS-CoV-2 vaccines and post-vaccination flares in patients with systemic lupus erythematosus. Vaccine.

[33] Gerosa M., Schioppo T., Argolini L.M., Sciascia S., Ramirez G.A., Moroni G., Sinico R.A., Bonelli G., Alberici F., Mescia F. (2022). The Impact of Anti-SARS-CoV-2 Vaccine in Patients with Systemic Lupus Erythematosus: A Multicentre Cohort Study. Vaccines.

[34] Louthrenoo W., Tangkum P., Kasitanon N., Gumtorntip W., Winichakoon P., Konsamun S., Wongthanee A. (2024). Flares and Predicting Factors of Flares in Patients with Systemic Lupus Erythematosus Associated with Different Doses and Types of COVID-19 Vaccines. Vaccines.

[35] Lisowska K.A., Ciesielska-Figlon K., Komorniczak M., Bułło-Piontecka B., Dębska-Ślizień A., Wardowska A. (2024). Peripheral Blood Mononuclear Cells and Serum Cytokines in Patients with Lupus Nephritis after COVID-19. Int. J. Mol. Sci..

