# Supplementary material for: Markers of Antiviral Response in SLE Patients After Vaccination Against SARS-CoV-2
Source: Int J Mol Sci. 2025 Oct 21;26(20):10241. doi: 10.3390/ijms262010241 (PMC12563841; doi:10.3390/ijms262010241)
Supplement: Supplementary file 1 [file ijms-26-10241-s001.zip › ijms-3933794-supplementary.pdf]

Supplementary material

Markers of antiviral response in SLE patients after vaccination against SARS-CoV-2

Supplementary Table S1. Gene-specific primers and probe sets (TaqMan™ Gene Expression Assay) used in the experiment for each lymphocyte population (T/B).

Gene symbol	Description	Assay ID	Population
GAPDH	Housekeeping gene	Hs02758991_g1	**T, B**
TBX21	T-bet (specific for Th1 subpopulation)	Hs00894392_m1	**T**
RORC	RORγt (specific for Th17 subpopulation)	Hs01076112_m1	**T**
GATA3	Transcription factor specific for the Th2 subpopulation	Hs00231122_m1	**T**
FOXP3	Transcription factor specific for Tregs	Hs01085834_m1	**T**
BAFF	B-cell-activating factor	Hs00198106_m1	**B**
TRIM21	Effector in the intracellular antibody-mediated proteolysis	Hs00172616_m1	**T**
ACE2	Angiotensin-converting enzyme type 2	Hs01085333_m1	**T**
APOBEC3G	Involved in antiretroviral activity against retroviruses	Hs00222415_m1	**T, B**
IRF8	Regulator of the expression of type I IFNs	Hs00175238_m1	**T, B**
ISG15	Protein induced by type I IFN acting as a cytokine	Hs00192713_m1	**T, B**
IFNG	The only member of the type II class of interferons	Hs00989291_m1	**T**
IL4	Cytokine, which induces differentiation into Th2 cells	Hs00174122_m1	**T**
IL6	Pro-inflammatory cytokine	Hs00174131_m1	**T, B**
TGFB	Anti-inflammatory cytokine	Hs00998133_m1	**T, B**
IL12	Cytokine, which induces differentiation into Th1 cells	Hs01073447_m1	**T, B**
IL2	Cytokine, which activates T cells	Hs00174114_m1	**T**
IL1A	Pro-inflammatory cytokine	Hs00174092_m1	**T**
IL17A	Pro-inflammatory cytokine	Hs00174383_m1	**T**
IL10	Anti-inflammatory cytokine	Hs00961622_m1	**T, B**
IL8	Pro-inflammatory chemokine, attracts neutrophils	Hs00174103_m1	**T**
TNF	Pro-inflammatory cytokine	Hs00174128_m1	**T, B**

Supplementary Table S2. T- and B-cell phenotype in SLE patients.

	**Fully vaccinated (*n* = 23)**	**Partially vaccinated (*n* = 10)**	***P* value**
**T-cell subpopulations**			
CD3+ [%]	72.90 (31.30; 90.20)	65.00 (25.60; 78.00)	0.060019
CD3+CD4+ [%]	34.10 (8.50; 77.30)	34.60 (12.00; 67.10)	0.916856
CD3+CD8+ [%]	52.50 (14.90; 80.10)	44.40 (27.40; 65.00)	0.205849
CD4+/CD8+	0.71 (0.11; 5.19)	0.63 (0.18; 46.30)	0.409926
CD4+CD28+ [%]	95.80 (51.70; 99.70)	95.70 (0.63; 99.40)	0.932633
CD8+CD28+ [%]	52.10 (23.10; 95.00)	67.15 (43.90; 93.30)	0.156884
CD4+CD69+ [%]	3.17 (0.07; 18.10)	4.07 (0.96; 70.30)	0.672573
CD8+CD69+ [%]	3.31 (0.05; 11.70)	3.16 (1.16; 9.82)	0.832646
CD4+HLA-DR+ [%]	7.16 (3.41; 16.50)	6.21 (1.72; 56.70)	0.446828
CD8+HLA-DR+ [%]	27.80 (2.78; 72.90)	18.70 (8.21; 39.10)	0.183107
CD4+CD197-CD45RA- [%]	13.90 (5.46; 40.40)	15.25 (6.18; 27.70)	0.767357
CD4+CD197+CD45RA- [%]	16.40 (6.66; 36.40)	20.10 (14.70; 31.30)	0.281129
CD4+CD197+CD45RA+ [%]	55.80 (18.00; 81.90)	52.95 (29.20; 69.20)	0.642016
CD4+CD197-CD45RA+ [%]	11.90 (1.73; 48.00)	9.93 (3.05; 42.10)	0.386336
CD8+CD197-CD45RA- [%]	3.14 (0.60; 17.40)	6.08 (2.04; 13.70)	0.075898
**CD8+CD197+CD45RA- [%]**	**1.15 (0.41; 6.66)**	**2.27 (1.45; 7.83)**	**0.009931**
CD8+CD197+CD45RA+ [%]	30.90 (11.80; 89.30)	40.05 (2.71; 55.00)	0.512387
CD8+CD197-CD45RA+ [%]	60.60 (9.07; 79.80)	49.10 (32.40; 62.30)	0.072474
CD4+CD127-CD25+ [%]	8.02 (2.75; 16.50)	9.27 (6.67; 57.00)	0.228457
**B-cell subpopulations**			
CD19+ [%]	6.22 (1.61; 15.60)	5.15 (1.34; 13.00)	0.949454
CD19+CD24++CD38++ [%]	0.89 (0.00; 9.88)	1.00 (0.00; 24.00)	0.703680
CD19+CD24-CD38++CD27+IgD- [%]	0.10 (0.00; 1.08)	0.41 (0.00; 6.87)	0.280789
CD19+CD27-IgD- [%]	9.64 (2.34; 28.50)	6.97 (1.35; 30.00)	0.320635
CD19+CD27+IgD- [%]	5.61 (1.51; 29.60)	9.17 (1.98; 15.20)	0.865759
CD19+CD27+IgD+ [%]	10.40 (1.76; 80.60)	11.39 (1.93; 87.10)	0.899110
CD19+CD27-IgD+ [%]	73.90 (4.38; 94.20)	67.55 (2.93; 87.60)	0.751271

Data are presented as median (minimum; maximum). Statistically significant results ($p < 0.05$) are shown in bold according to the U Mann-Whitney test. **Abbreviations:** CD, cluster of differentiation; HLA-DR, human leukocyte antigen–DR isotype; IgD, immunoglobulin D.

Supplementary Table S3. T- and B-cell gene expression in SLE patients.

	Fully vaccinated	Partially vaccinated	P value
**T-cell gene expression**			
TBX21	0.047 (0.007; 0.141)	0.036 (0.010; 0.108)	0.279941
RORC	0.008 (0.001; 0.033)	0.013 (0.003; 0.021)	0.487387
GATA3	0.118 (0.018; 0.215)	0.089 (0.044; 0.225)	0.395994
FOXP3	0.033 (0.003; 0.079)	0.027 (0.015; 0.045)	0.425245
TRIM21	0.044 (0.004; 0.070)	0.033 (0.019; 0.051)	0.236670
ACE2	0.000 (0.000; 0.000)	0.000 (0.000; 0.000)	0.335832
APOBEC3G	0.360 (0.174; 0.676)	0.304 (0.054; 0.422)	0.315727
IRF8	0.010 (0.003; 0.029)	0.014 (0.006; 0.020)	0.157105
**ISG15**	**0.023 (0.008; 0.063)**	**0.010 (0.005; 0.061)**	**0.028770**
IFNG	0.007 (0.000; 0.024)	0.005 (0.003; 0.016)	0.395922
IL-4	0.000 (0.000; 0.000)	0.000 (0.000; 0.000)	0.353731
IL-6	0.000 (0.000; 0.000)	0.000 (0.000; 0.001)	0.156915
TGFB	1.313 (0.323; 2.035)	1.231 (0.862; 1.784)	0.857115
IL-12	0.001 (0.001; 0.003)	0.001 (0.000; 0.002)	0.075939
IL-2	0.001 (0.000; 0.001)	0.000 (0.000; 0.002)	0.897670
IL-1A	0.000 (0.000; 0.000)	0.000 (0.000; 0.000)	0.184181
IL-17	0.000 (0.000; 0.000)	0.000 (0.000; 0.000)	0.962421
IL-10	0.000 (0.000; 0.002)	0.000 (0.000; 0.001)	0.661875
IL-8	0.002 (0.000; 0.229)	0.012 (0.000; 0.250)	0.236670
TNF	0.006 (0.001; 0.011)	0.006 (0.005; 0.013)	0.757549
**B-cell gene expression**			
APOBEC3G	0.215 (0.009; 0.534)	0.104 (0.062; 0.310)	0.113161
IRF8	0.092 (0.002; 0.787)	0.074 (0.01;3 0.514)	0.221688
ISG15	0.008 (0.000; 0.081)	0.009 (0.000; 0.028)	0.927872
BAFF	0.117 (0.003; 0.363)	0.215 (0.048; 1.053)	0.160588
IL6	0.007 (0.000; 0.022)	0.005 (0.000; 0.015)	0.333557
TGFB	0.930 (0.000; 1.653)	0.593 (0.000; 3.364)	0.650719
IL12	0.002 (0.000; 0.017)	0.003 (0.000; 0.010)	0.688646
IL10	0.000 (0.000; 0.016)	0.00 0(0.000; 0.006)	0.440613
TNF	0.004 (0.000; 0.011)	0.006 (0.000; 0.013)	0.571339

Data are presented as median (minimum; maximum). Statistically significant results ($p < 0.05$) are shown in bold according to the U Mann-Whitney test. **Abbreviations:** BAFF, B-cell activating factor; IFN, interferon; IL, interleukin; IRF8, interferon regulatory factor 8; ISG15, interferon-stimulated gene 15; RORC, RAR-related orphan receptor C; TBX21, T-box transcription factor 21; TGFB, transforming growth factor-β; TNF, tumor necrosis factor; TRIM21, tripartite motif containing 21

Supplementary Table S4. Correlations of disease activity/damage indices and serologic markers with cytokine and SARS-CoV-2 neutralizing antibody responses in SLE.

**SLEDAI**	**R**	**t(N-2)**	**P value**
IL-6 [pg/mL]	0.388824	2.702332	0.009971
**SDI**	**R**	**t(N-2)**	**p**
IL-8 [pg/mL]	0.426657	2.565678	0.016039
GM-CSF [pg/mL]	0.356639	2.028210	0.051704
IFN-β	0.427706	2.596599	0.015280
IL-10	0.422750	2.556418	0.016589
Alpha Neut. Ab.	0.427003	2.629199	0.013200
**C3**	**R**	**t(N-2)**	**p**
IFN-α2	-0.444982	-2.765505	0.009356
IFN-γ	-0.361553	-2.100046	0.043057
**C4**	**R**	**t(N-2)**	**p**
Alpha Neut. Ab.	0.363775	2.174393	0.037425
Omicron Neut. Ab.	0.471181	2.974228	0.005604
**IgG**	**R**	**t(N-2)**	**p**
IP-10	0.592350	3.968084	0.000436
**Anti-dsDNA [IU/mL]**	**R**	**t(N-2)**	**p**
IP-10	0.531342	3.377661	0.002058
Omicron Neut. Ab.	-0.501464	-3.121283	0.004005

Spearman rank-order correlations between clinical/serologic indices and immune readouts are shown. Only statistically significant associations ($p < 0.05$) are listed; values denote correlation coefficient (R), t statistic [t(N−2)], and two-sided p-value. **Abbreviations**: SLEDAI, Systemic Lupus Erythematosus Disease Activity Index; SDI, SLICC Damage Index; Neut. Ab., neutralizing antibody; GM-CSF, granulocyte–macrophage colony-stimulating factor; IFN, interferon; IL, interleukin; TNF, tumor necrosis factor; dsDNA, double-stranded DNA; IP-10, interferon-γ–induced protein-10.
